# How has body weight changed since the beginning of the COVID-19 pandemic?

**DOI:** 10.25646/10670

**Published:** 2022-12-20

**Authors:** Anja Schienkiewitz, Stefan Damerow, Almut Richter, Gert B.M. Mensink

**Affiliations:** Robert Koch Institute, Berlin Department of Epidemiology and Health Monitoring

**Keywords:** WEIGHT CHANGE, WEIGHT GAIN, COVID-19 PANDEMIC, ADULTS, GEDA 2021

## Abstract

**Background:**

Measures for containing the COVID-19 pandemic in 2020 and 2021 resulted in drastic changes in physical activity and dietary habits that also impacted body weight.

**Methods:**

The representative study German Health Update (GEDA 2021) includes self-reported information about body weight and body height for adults aged 18 years and older (n=2,985) from July to October 2021. In addition, the study asked about changes in body weight since the beginning of the COVID-19 pandemic.

**Results:**

For 59% of participants, body weight has not changed since the beginning of the COVID-19 pandemic, 26% report weight gain, and 15% report weight loss. Younger people indicate weight gain more often than older people, and individuals with obesity report weight gain more often than individuals without obesity. 1.5 years after the beginning of the COVID-19 pandemic, the average weight change within the population is approximately +0.34kg.

**Conclusions:**

The effects of restrictions in everyday life with regard to the possible negative impacts on body weight should be given greater consideration and should be monitored in the future.

## Introduction

The measures for containing the COVID-19 pandemic in 2020 and 2021 resulted in drastic changes in the lifestyle and wellbeing of many people. There are indications that changes in physical activity and dietary habits manifest in the course of the COVID-19 pandemic and lead to weight changes [1–3]. Initial results from Germany from survey data for adults were available in September 2020. At that time, approximately 1,000 parents between the ages of 20 and 65 with children up to the age of 14 were asked about weight changes since the beginning of the COVID-19 pandemic. 27% indicated having gained weight since the beginning of the COVID-19 pandemic [4]. An evaluation of the study German Health Update (GEDA 2019/2020-EHIS) showed an increase of the average body weight by 1.1kg and an increase of the average BMI by 0.5kg/m^2^ [5] in a comparison between the pre-pandemic period April to August 2019 and April to August 2020. A more recent evaluation suggests that this increase did not continue after October 2020 [6]. In such a population-wide observation, individual changes of weight gain and of weight loss balance each other out to some extent. Therefore, an analysis will be made here, based on survey data across Germany, to find out which groups of people have been affected by weight changes since the beginning of the COVID-19 pandemic, and how many kilograms they have gained or lost. The data for this was acquired between July and October 2021, and thus approximately 1.5 years after the beginning of the COVID-19 pandemic.

**GEDA 2021**

Sixth follow-up survey of the German Health Update

**Data holder:** Robert Koch Institute

**Objectives:** Provision of reliable information on the health status, health behaviour and health care of the population living in Germany and their changes in the course of the SARS-CoV-2 pandemic.

**Study design:** Cross-sectional telephone survey

**Population:** German-speaking population aged 16 years and older living in private households that can be reached via landline or mobile phone

**Sampling:** Random sample of landline and mobile telephone numbers (dual-frame method) from the ADM sampling system (Arbeitskreis Deutscher Markt- und Sozialforschungsinstitute e.V.)

**Sample size:** 5,030 respondents

**Study period:** July 2021 to December 2021

**GEDA survey waves:**

**►** GEDA 2009
**►** GEDA 2010
**►** GEDA 2012
**►** GEDA 2014/2015-EHIS
**►** GEDA 2019/2020-EHIS
**►** GEDA 2021

Further information in German is available at www.geda-studie.de

## Indicator

The German Health Update (GEDA) is a cross-sectional survey of the residential population living in Germany, which has the objective of describing the state of health, the health care, and the health behaviour, and of capturing demographic and socioeconomic determinants. GEDA 2021 is a telephone survey of people aged 16 years and older.

From July to October, participants were asked about body height and weight, and the body mass index (BMI, kg/m^2^) was calculated from this information. In addition, the question ‘Has your body weight changed since the beginning of the Corona pandemic, thus since March 2020?’ was asked. The response options were: ‘Yes, I gained weight”, ‘yes, I lost weight’, and ‘no, remained the same’. Participants who reported weight gain or loss were then asked for an estimate in kg (question: ‘Approximately how many kilograms?’).

The analyses are based on information from 2,985 participants aged 18 years and older. Valid information relating to weight changes during the pandemic is available for 2,965 individuals. Evaluations relating to the average weight change in kilograms are based on valid information from 2,944 individuals who report a weight gain or loss (n=1,114). Individuals whose body weight remained the same (n=1,830) are assigned the value 0kg for the weight change.

Prevalence was reported with 95% confidence intervals (95% CI) by gender (women, men) [7], age group (aged 18–29, aged 30–44, aged 45–64, ≥65 years of age) and education group (International Standard Classification of Education, ISCED: Low, medium, high education group) [8], and mean values (M) were identified with 95% CI. In a multinominal logistic regression (outcome: gain/loss, reference: same/stable weight), gender, age, education, and obesity (BMI≥30kg/m^2^) were included, and odds ratios (OR) were calculated as effect estimates with 95% confidence intervals (95% CI). An OR can be interpreted as the factor, by which the odds of an event increases (here, e.g., weight gain), when being exposed to the risk factor. Only participants with valid values in all variables (n=2,909) are taken into consideration for the regression analysis.

To correct deviations of the sample from the population structure, the analyses were performed using a weighting factor. First of all, a design weighting was performed for the various selection probabilities (mobile service and landline) as part of the data weighting. Then, an adaptation to the official population numbers was made based on age, sex, federal state, district type (as of: 31.12.2020), and education (microcensus 2018). All analyses were performed using SAS 9.4. A significant difference between the groups is assumed when the p-value, which is calculated in consideration of the weighting and of the survey design, is less than 0.05.

## Results and discussion

Almost 60% of the participants indicate that their body weight remained the same since the beginning of the COVID-19 pandemic, 26.2% report weight gain, and 14.5% report weight loss. There were no statistically significant differences between men and women or between education groups. Significant differences can be observed with age and the BMI. Older people report unchanged body weight more often than younger people. In contrast, younger people indicate weight gain more often than older people. This is also observed among middle-aged groups. On average, those with unchanged weight are eight years older than those with weight gain. With 27.3kg/m^2^ among participants who indicate weight gain, the average BMI is significantly higher compared to those who report weight loss (25.5kg/m^2^) or stable weight (25.3kg/m^2^). Obesity is present among 47.8% of those whose body weight remained the same, among 39.1% of those who indicate weight gain, and among 13.1% of those who indicate weight loss (Table 1).

The multinominal regression analysis shows a higher odds ratio for women to have gained body weight since the beginning of the COVID-19 pandemic. In addition, age is a further determinant for a weight change: The younger the individuals, the higher the odds ratio of weight gain. It also is observed that people in the youngest age group compared to those aged 65 years and older indicate weight loss more frequently. Obesity is associated with a higher odds ratio for weight gain (Table 2).

The average weight change within the population since the beginning of the COVID-19 pandemic is +0.34kg (95% CI: 0.07–0.61). Among those who have indicated weight gain since the beginning of the COVID-19 pandemic, the average weight gain is 5.3kg (95% CI: 4.8–5.8). Those who report weight loss, have lost 7.0kg (95% CI: 6.3–7.7) on average.

The GEDA 2021 study provides population-based survey data from July to October 2021 on the subjective weight change, which, in retrospect, includes a period of approximately 1.5 years since the beginning of the COVID-19 pandemic. During that time, temporary containment measures led to long-lasting restrictions in everyday life, such as increased sedentary activities and less physical activity [9]. With regard to the development of the body weight, the odds ratio for weight gain was increased especially for younger people and individuals with obesity.

A placement of the available results of the study in the existing national and international literature on weight change since the beginning of the COVID-19 pandemic is only possible to a very limited extent because not only the times of the survey and thus also associated different infection dynamics and restrictions in everyday life vary greatly across individual countries, but also because survey methods and the wording of questions differ as well. In addition, the methodological quality of the studies is very heterogeneous and the validity may be limited due to lack of representativeness (e.g. selected samples in social media). Moreover, many studies – such as this GEDA 2021 study – are cross-sectional studies, which ask retrospectively about weight changes in the beginning of the COVID-19 pandemic, and this information can be biased by personal recollection.

A rapid review analysed the impact of the containment and quarantine measures on modifiable cardiovascular risk factors within populations and draws the conclusion that at least one fourth of adults reports weight gain [1]. In an online survey conducted in April 2020 in the USA, 27.5% of participants also indicated weight gain, even 33.4% among individuals with obesity [10], a magnitude, which was also found in this GEDA 2021 study. In a representative online survey in April 2021 among adults between the ages of 18 and 70 years in Germany, 48% of participants indicated no weight change, 39% indicated weight gain, and 11% indicated weight loss since the beginning of the COVID-19 pandemic [11]. A repeated survey in May/June 2022 showed that 49% of participants had no change in weight, 35% reported weight gain, and 15% reported weight loss since the beginning of the COVID-19 pandemic [12]. With 15%, the proportion of those who reported in GEDA 2021 after 1.5 years having lost weight is identical, and the proportion without weight changes is significantly greater with 59%. The nu3 Corona study, which was conducted in April 2020 [13], already showed that younger people indicate weight gain more frequently than older people. Individuals between the ages of 35 and 44 years reported weight gain most frequently (29%), in the online survey from April 2021 it was individuals between the ages of 30 and 44 years [11]. The fact that individuals with obesity report weight gain significantly more frequently was also the result of an online survey in the USA [10]. In GEDA 2021, the average weight gain since the beginning of the COVID-19 pandemic was 340g. This is thus slightly higher than the average weight gain per year, observed in a longitudinal evaluation of cohort studies in Germany between 1994 and 2007. At that time, the average weight gain within the general population between the ages of 45 and 64 was 250g in men, and 240g in women [14]. The change is similar to the one already described earlier for Western countries in the period between mid-November and mid-January [15]. A weight gain of 340g in just under 1.5 years is probably clinically insignificant, but there are substantial deviations from this average value. Among those who indicate weight gain or loss, respectively, the average change of +5kg or -7kg, respectively, lies within the range of the online survey from April 2021 and May/June 2022 [11, 12]. If these weight changes observed since the beginning of the COVID-19 pandemic continue, impacts on the health of the population cannot be ruled out. For example, weight gain in women between the ages of 40 and 55 is associated with significantly higher odds of multimorbidity [16].

Even though only subjective estimates considering changes in body weight are available in the GEDA 2021 study, and comparatively acquired data from the pre-pan-demic period is missing, the results represented here show that restrictions in everyday life caused by the COVID-19 pandemic have possibly influenced the body weight in the last 1.5 years. Certain population groups, such as younger people and individuals with obesity, were affected more frequently by weight changes. In the long run, (persistent) weight gain can go hand in hand with health risks and other non-communicable diseases, which are associated with overweight and obesity. This is why the effects of restrictions in everyday life should be given greater consideration with regard to the possible negative impacts on body weight and should be monitored in the future.

## Key statements

- 59% of participants indicate that their body weight remained the same since the beginning of the COVID-19 pandemic, 26% report weight gain, and 15% report weight loss.
- Younger people report weight gain more often than older people.
- People with obesity report weight gain more often than people without obesity.
- The average weight change within the population around 1.5 years after the beginning of the COVID-19 pandemic is +0.34kg.

## Figures and Tables

**Table 1:** Subjective change of body weight since the beginning of the COVID-19 pandemic (N=2,965, n=1,421 women, n=1,544 men) by gender, age, education, and body mass index Source: GEDA 2021

	%	Stable weight n=1,830	%	Weight gain n=747	%	Weight loss n=388	p-value*
		(95% CI)		(95% CI)		(95% CI)	
**Total**	**59.3**	**(56.5–62.0)**	**26.2**	**(23.8–28.7)**	**14.5**	**(12.6–16.7)**	**<0.0001**
**Gender**							0.0724
Women	56.2	(52.4–59.9)	27.5	(24.2–31.1)	16.3	(13.5–19.5)	
Men	62.3	(58.3–66.2)	24.9	(21.6–28.6)	12.8	(10.3–15.8)	
**Age (years, M)**	54.4	(52.9–55.9)	46.3	(44.4–48.3)	49.4	(46.2–52.7)	<0.0001
**Age group**							<0.0001
18–29 years	46.5	(38.2–55.0)	35.1	(27.6–43.5)	18.4	(12.4–26.5)	
30–44 years	53.3	(47.2–59.4)	30.7	(25.4–36.7)	15.9	(11.9–21.1)	
45–64 years	61.5	(57.2–65.6)	27.4	(23.7–31.3)	11.2	(8.7–14.2)	
≥65 years	69.4	(65.1–73.5)	15.5	(12.5–18.9)	15.1	(12.1–18.7)	
**Education status**							0.3023
Low education group	66.2	(57.1–74.2)	20.3	(13.8–28.8)	13.5	(8.4–20.8)	
Medium education group	57.6	(53.9–61.2)	27.3	(24.1–30.7)	15.2	(12.6–18.1)	
High education group	59.0	(55.3–62.6)	27.1	(24.0–30.5)	13.8	(11.1–17.1)	
**BMI (kg/m^2^, M)**	25.3	(25.0–25.6)	27.3	(26.7–27.8)	25.5	(24.8–26.2)	<0.0001
**Obesity (BMI≥30kg/m^2^)**	47.8	(41.2–54.5)	39.1	(32.7–45.9)	13.1	(9.3–18.1)	<0.0001

^*^ p-value: group differences

CI = confidence interval, M = mean, BMI = body mass index

**Table 2:** Multinominal logistic regression for weight change^◊^. Odds ratios by gender, age, education, and body mass index (n=1,510 women, n=1,399 men) Source: GEDA 2021

	Weight gain OR (95% CI)		Weight loss OR (95% CI)	
**Gender**				
Women	1.33	(1.02–1.75)^*^	1.38	(0.98–1.96)
Men		1.0		1.0
**Age group**				
18–29 years	4.16	(2.61–6.63)^**^	1.88	(1.05–3.37)^*^
30–44 years	2.74	(1.86–4.03)^**^	1.47	(0.94–2.29)
45–64 years	1.95	(1.40–2.71)^**^	0.86	(0.58–1.26)
≥65 years		1.0		1.0
**Education status**				
Low education group		1.0		1.0
Medium education group	1.61	(0.95–2.71)	1.38	(0.77–2.47)
High education group	1.64	(0.96–2.78)	1.30	(0.71–2.38)
**Obesity** **(BMI≥30kg/m^2^)^◊◊^**	2.52	(1.79–3.55)^**^	1.25	(0.80–1.96)

* p<0.05

** p<0.001

*◊* reference=category ‘stable weight’

^◊◊^ reference=no obesity

OR = odds ratio, CI = confidence interval, BMI = body mass index
